# Structure prediction of novel isoforms from uveal melanoma by AlphaFold

**DOI:** 10.1038/s41597-023-02429-z

**Published:** 2023-08-04

**Authors:** Zhe Zhang, Chen Li, Qian Li, Xiaoming Su, Jiayi Li, Lili Zhu, Xinhua (James) Lin, Jianfeng Shen

**Affiliations:** 1 Department of Ophthalmology, Ninth People’s Hospital, Shanghai Jiao Tong University School of Medicine, Shanghai, 200025 China; 2 Shanghai Key Laboratory of Orbital Diseases and Ocular Oncology, Shanghai, 200025 China; 3 Institute of Translational Medicine, National Facility for Translational Medicine, Shanghai Jiao Tong University, Shanghai, 200240 China; 4 High Performance Computing Center, Shanghai Jiao Tong University, Shanghai, 200240 China; 5 State Key Laboratory of Microbial Metabolism, Joint International Research Laboratory of Metabolic & Developmental Sciences, School of Life Sciences & Biotechnology, Shanghai Jiao Tong University, Shanghai, 200240 China; 6 Songjiang Research Institute and Songjiang Hospital, Shanghai Jiao Tong University School of Medicine, Shanghai, 201600 China

**Keywords:** Computational biology and bioinformatics, Diseases

## Abstract

Alternative splicing is an important mechanism that enhances protein functional diversity. To date, our understanding of alternative splicing variants has been based on mRNA transcript data, but due to the difficulty in predicting protein structures, protein tertiary structures have been largely unexplored. However, with the release of AlphaFold, which predicts three-dimensional models of proteins, this challenge is rapidly being overcome. Here, we present a dataset of 315 predicted structures of abnormal isoforms in 18 uveal melanoma patients based on second- and third-generation transcriptome-sequencing data. This information comprises a high-quality set of structural data on recurrent aberrant isoforms that can be used in multiple types of studies, from those aimed at revealing potential therapeutic targets to those aimed at recognizing of cancer neoantigens at the atomic level.

## Background & Summary

Alternative splicing (AS) can influence transcriptome and proteome diversity, as evidence shows that approximately 95% of genes with multiple exons produce multiple isoforms^1,2^. Therefore, it is not surprising that the gene isoforms play important roles in many biological processes, such as processes related to development, pluripotency and apoptosis^3–5^. Aberrant isoforms have been implicated in multiple human tumors, including uveal melanoma (UM), showing extensive changes via alternative splicing and the expression of critical gene isoforms^6–8^. Specific splicing isoforms are important for the initiation, metastasis and drug resistance of cancer, and some AS events have been shown to be significantly related to patient survival^9–11^. Although the role of a few splicing isoforms in cancer has been studied, 3D protein structure prediction on a scale that covers the transcriptome and can be used for evaluating biological functionality remains unexplored.

The suitability of short-read mRNA sequencing (short-read RNA-seq) in the discovery of AS events is limited because of the mapping uncertainty of short read lengths or assembly problems^12^. Long-read mRNA sequencing (long-read RNA-seq) shows advantages over short-read RNA-seq in isoform detection because long reads directly cover the entire transcript without the need of reconstruction, which is needed for short reads^13–15^. However, because of the high sequencing error rate (~15%) of raw long-read RNA-seq data, it is still challenging to determine the precise splicing sites with only long-read RNA-seq data^16^. Hybrid sequencing, combining long-read RNA-seq reads with high quality short-read RNA-seq reads and taking advantage of both platforms, improves the identification of AS events and gene isoforms^17,18^. Although great efforts have been made to study the alternative splicing mechanisms and functions of different isoforms, our knowledge of the 3D structure of splicing isoforms is very limited^19,20^. This lack of information means that for a large majority of spliced isoforms, no documented structures have been deposited in the Protein Data Bank (PDB), causing a large knowledge gap; hence, accurate prediction of protein structure is one of the most challenging goals in biology^21,22^. As structures carry vital information about how different isoforms with a certain degree of sequence homology perform different functions, it is necessary to investigate the 3D structure of abnormal isoforms to explore their functions^23^. The most recent achievement in related technology, AlphaFold, a deep-learning-based approach, has been proven to be highly successful in predicting the 3D structures of proteins based on their amino acid sequences^24^. This is a significant advance that might have a profound impact on the study of protein dysfunction and the discovery of new polypeptides with potential medical applications^25^.

In this study, we provide an information resource based on the predicted structures of 315 novel isoforms obtained by long-read RNA-seq and short-read RNA-seq of transcriptome data from 18 UM patients. To better understand the structural differences of abnormal isoforms and the potential effects, we compared the structural differences between 295 abnormal-gene-encoded isoforms and their normal gene-encoded protein counterparts. We also identified 13 potential AS-derived neoantigens in 10 abnormal isoforms with altered amino acid sequences. These data constitute particularly valuable information on aberrant isoform structures that intersects with that on abnormal isoforms in other datasets, which can be used for an investigation into the roles of these isoforms in multiple cancer types. This study also offers new insights into the structure-based prediction of neoantigens and potential drug targets.

## Methods

### Patient samples

A total of 20 patients with primary UM who visited Shanghai Ninth People’s Hospital between 2018 and 2021 were selected for sampling. The detail information of the 20 UM patients is listed in Table 1. The process of sample collection adhered to the tenets of the Declaration of Helsinki and was approved by the Ethics Committee of Shanghai Ninth People’s Hospital, Shanghai Jiao Tong University School of Medicine (SH9H-2021-T82-1). All patient samples were donated freely, with written informed consent and with the full cooperation of each patient. We have confirmed that in our ethics statement, patients consent to the disclosure of genomic data. The critical exclusion criteria include previous treatment of chemotherapy or radiotherapy. Sterilized instruments collected the samples, and immediately underwent snap-frozen in liquid nitrogen and stored at a temperature below −80 °C.

**Table 1:** The detail information of UM patients.

Patient	Age (yrs)	Gender (male/female)	Ethnicity	Largest basal diameter*thickness (mm)
Case 1	30–35	male	Chinese	18.55*8.54
Case 2	30–35	male	Chinese	19.53*10.67
Case 3	45–50	male	Chinese	12.98*7.78
Case 4	45–50	female	Chinese	14.07*6.59
Case 5	45–50	male	Chinese	18.28*9.53
Case 6	45–50	male	Chinese	11.65*9.19
Case 7	45–50	male	Chinese	10.58*8.66
Case 8	45–50	female	Chinese	12.43*11.25
Case 9	50–55	male	Chinese	11.95*8.12
Case 10	50–55	male	Chinese	13.22*14.42
Case 11	50–55	female	Chinese	16.64*9.83
Case 12	50–55	female	Chinese	18.9*13.4
Case 13	55–60	female	Chinese	15.06*10.07
Case 14	55–60	male	Chinese	14.83*6.63
Case 15	65–70	female	Chinese	14.7*8.83
Case 16	65–70	male	Chinese	10.5*5.02
Case 17	70–75	male	Chinese	15.23*7.79
Case 18	70–75	male	Chinese	10.01*11.13
Case 19	70–75	male	Chinese	15.95*5.25
Case 20	75–80	male	Chinese	12.25*5.15

### Illumina sequencing

Total RNA was isolated using Trizol Reagent (Invitrogen Life Technologies), then the concentration, quality and integrity of RNA were determined by NanoDrop spectrophotometer (Thermo Scientific). Three micrograms of RNA were used as input material for the RNA sample preparations. Sequencing libraries were generated which was then sequenced on Illumina NovaSeq. 6000 platform by Shanghai Personal Biotechnology Cp. Ltd.

### Nanopore sequencing

Total RNA was isolated using the Trizol Reagent (Invitrogen Life Technologies), and the concentration, quality and integrity were determined by NanoDrop spectrophotometer (Thermo Scientific). A total of 1ug RNA was prepared for cDNA libraries using the cDNA-PCR Sequencing Kit (SQK-PCS109) according to the instructions of Nanopore Technologies (ONT). Defined PCR adaptors were directly added to both ends of the first-strand cDNA by reverse transcriptase. After 14-cycle of PCR by LongAmp Tag (NEB), the PCR products were subjected to ONT adaptor ligation using T4 DNA ligase (NEB). Agencourt XP beads were used for DNA purification according to ONT protocol. The final cDNA libraries were added to FLO-MIN109 flow cells and were sequenced on the PromethION platform. GUPPY (version 3.2.6) was used for basecalling to convert the fast5 format data to fastq format.

### Hybrid-sequencing strategy

**Fig. 1:**
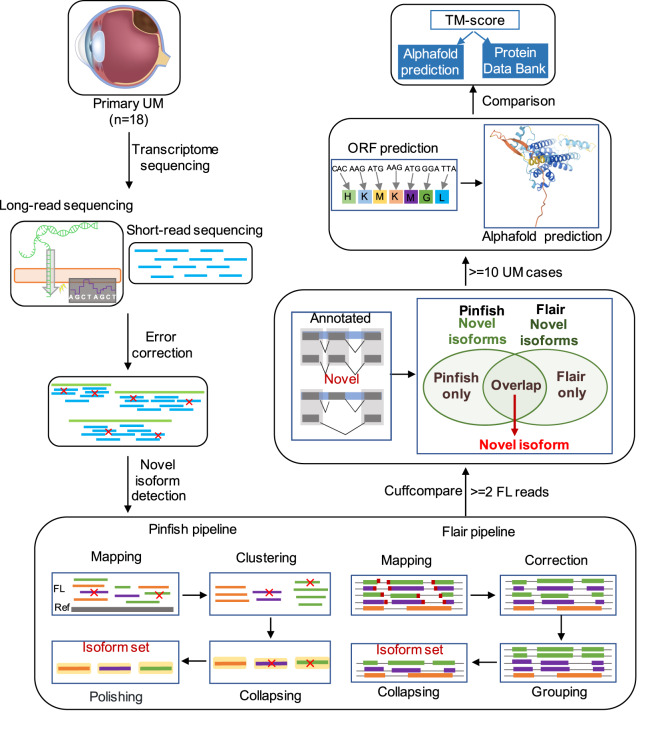
The overall workflow of this study.

Hybrid error correction, a simple and cost-effective approach involved with high quality short-read RNA-seq data, was used to improve the quality of long reads (Fig. 1). Here we used LoRDEC to correct the errors of full-length (FL) sequences^17^. LoRDEC is a new and efficient hybrid correction algorithm based on De Bruijn Graphs (DBG) of short reads. Achieving a comparable accuracy, LoRDEC runs six times faster and requires 93% less memory than PacBioToCA and LSC. LoRDEC first reads the short reads, builds their DBG of order k and then corrects each long read one after the other independently.

### Pinfish pipeline

The corrected FL reads for each sample were aligned to hg38 using minimap2 with the command ‘minimap2 -ax splice’^26^. Spliced_bam2gff was used to convert sorted BAM files with spliced alignments (from minimap2) into GFF2 format. With sorted GFF2 file as input, based on the median of exon boundaries from all transcripts in the cluster, cluster_gff clusters reads with similar exon/intron structures into a rough consensus set of clusters. Then, by mapping all reads to the median length of read within each cluster generated by cluster_gff, polish_clusters creates an error corrected read and polishes it using racon^27^. Finally, taking polished and consistent transcripts as input, collapse_partials filters transcripts which are likely caused by 5′ end degradation and collapses input transcripts into a polished and collapsed transcripts set of each UM case (Fig. 1).

### Flair pipeline

Flair_align aligns FL reads of each sample to hg38 using minimap2 and converts the SAM output to BED format. Flair_correct corrects mis-aligned splice sites with genome annotations and short-read splice junctions generated by STAR^28^. Finally, flair_collapse defines high-confidence transcript sets from corrected long reads^14^ (Fig. 1).

### Novel isoform detection

**Fig. 2:**
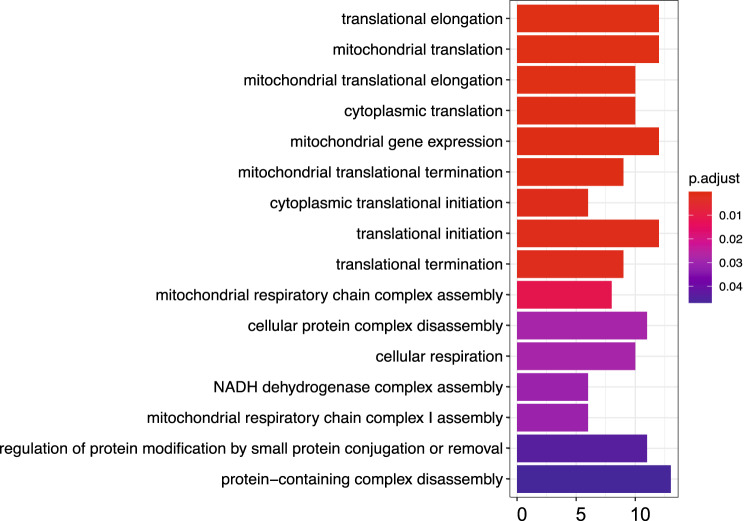
Gene ontology enrichment of genes with novel isoforms.

We filtered out transcripts supported by less than two FL reads. With the cuffcompare tool in the Cufflinks package^29^, we compared the high-confidence isoforms output by flair and pinfish pipelines with the “RefSeq” gene annotation file, respectively. Cuffcompare explores the structure of each isoform, and matches reference transcripts that agrees on the coordinates and orders of all their exons, as well as strand. Isoforms set were further classified into eight groups based on their exon structures (splicing junctions) after the cuffcompare process. Isoform labeled by “ = ” and “j” tags in the output “.tracking” file was considered as an annotated and unannotated (novel) isoform, respectively. We got a median of 8,989 annotated and a median of 9,150 novel isoform candidates based on pinfish pipeline (Table 2), with a median of 11,117 annotated and a median of 12,366 novel isoform candidates from flair pipeline (Table 2). Finally, we defined novel isoforms as those were identified as novel isoform candidates in both flair and pinfish pipelines of each case. In order to further define high-confidence and recurrent novel isoform set for further analysis, we only remained 315 novel isoforms which have been identified in more than 10 UM cases (Fig. 1). We then performed GO enrichment analysis and found regulation of translation initiation, elongation and termination related pathways were significantly enriched in these isoform related genes (Fig. 2).

**Table 2:** Statistics of annotated and novel isoforms among 18 UM cases.

Case	Annotated		Novel candidates		Novel
	Pinfish	Flair	Pinfish	Flair	
Case 2	7118	8622	3074	4905	1020
Case 3	10004	12655	15696	20561	3956
Case 4	11702	14581	15639	20352	4500
Case 5	5678	7818	4074	6866	1141
Case 6	9599	11606	10321	13437	3601
Case 7	11428	13740	15518	17572	4335
Case 9	7523	9571	11110	11451	2788
Case 10	6724	8477	3237	5270	958
Case 11	8901	11153	9144	13214	3041
Case 12	10893	13317	12302	16035	3718
Case 13	4031	6114	6591	11919	1552
Case 14	9185	11081	9157	11289	2565
Case 15	11062	13508	14755	23147	4235
Case 16	2599	4298	4602	6847	1010
Case 17	9077	12108	8052	12814	2522
Case 18	12400	14773	15879	21276	4934
Case 19	8444	10813	7038	10729	2687
Case 20	3041	4873	7208	10365	1726

### Alphafold structure prediction

For novel isoforms, we first extracted corresponding DNA sequences from the human reference genome (hg38) using “samtools faidx” based on the coordinates and orders of all their exons, as well as the strand^30^. The tool of ORFfinder (https://www.ncbi.nlm.nih.gov/orffinder/) was subsequently employed to search for open reading frames (ORFs)^31^. The 3D structure was predicted using AlphaFold-Multimer version 2.2.0 using Shanghai Jiao Tong University’s supercomputing resources (more specifically, one NVIDIA Volta V100 GPUs with 32GB graphics processing unit (GPU) memory)^24^. The version and parameters of AlphaFold-Multimer databases used were outlined as below:

“python run_alphafold.py

--use_gpu_relax

--data_dir = $DIR

--uniref90_database_path = $DIR/uniref90/uniref90.fasta

--mgnify_database_path = $DIR/mgnify/mgy_clusters_2018_12.fa

--bfd_database_path = $DIR/bfd/bfd_metaclust_clu_complete_id30_c90_final_seq.sorted_opt

--uniclust30_database_path = $DIR/uniclust30/uniclust30_2020_06/UniRef30_2020_06

--pdb_seqres_database_path = $DIR/pdb_seqres/pdb_seqres.txt

--template_mmcif_dir = $DIR/pdb_mmcif/mmcif_files

--obsolete_pdbs_path = $DIR/pdb_mmcif/obsolete.dat

--uniprot_database_path = $DIR/uniprot/uniprot.fasta

--model_preset = multimer

--max_template_date = 2022-1-1

--db_preset = full_dbs

--output_dir = output

--fasta_paths = input.fasta”

### TM-score calculation for structural comparison

**Fig. 3:**
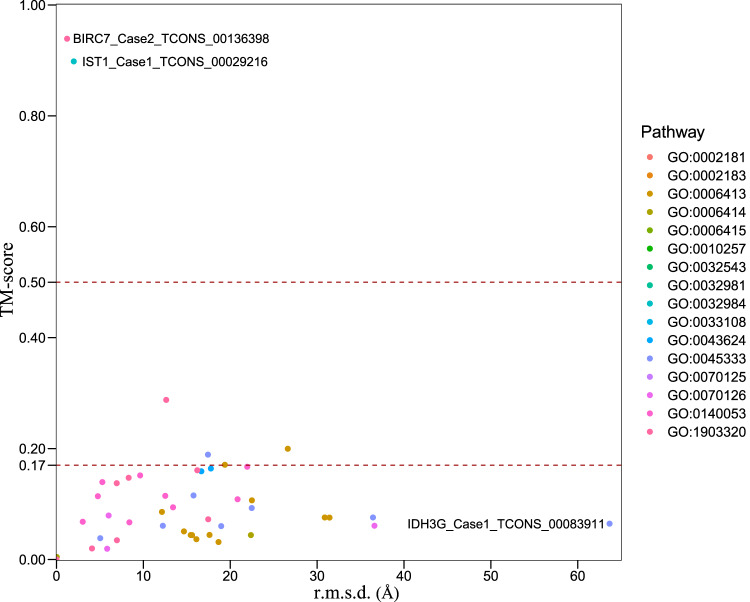
Structure comparison of novel isoforms with typical proteins.

Typical structure files were downloaded from Uniprot (https://www.uniprot.org) according to the priority order of EM, NMR, X-ray and alphafold predicted sources of structures. We used TM-score (https://zhanggroup.org/TM-score) to compare the predictive results with typical structures^32^. Protein pairs with a TM-score > 0.5 are mostly in the same fold while those with a TM-score < 0.5 are mainly not in the same fold, and some of them with a TM-score < 0.17 just have random structural similarity^33^. We then check the distribution of comparison scores of novel isoforms based on the gene ontology enrichment results above (Fig. 3). As only 32 pairs (32/295) of both ratios of aligned length to novel protein and aligned length to canonical protein are bigger than 0.9, which suggested that most of novel isoforms have random or low structures similarities with their canonical proteins. TM-score results of each paired comparison were deposited at figshare^34^.

### Neoantigens prediction

**Fig. 4:**
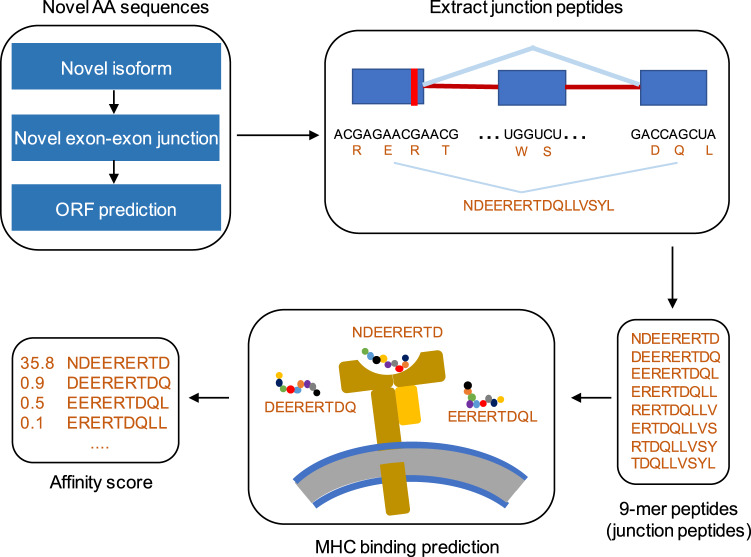
Overview of the neojunction-derived putative neoantigens detection.

For predicting AS-derived neoantigens, we first used a custom script to extract the unannotated splicing site information from all novel isoforms and defined such splicing junctions as neojunctions. Based on neojunction loci, we obtained all polypeptides generated by a neojunction. We used a custom script to extract all possible 9-amino acid sequences from these polypeptides. NetMHCPan was used to perform MHC-I binding affinity prediction. NetMHCpan methods inform if a sequence is a strong MHC binder if the % Rank is below the specified threshold (0.5%), and define the peptide as a weak binder if the % Rank is above the threshold of the strong binders but below the specified threshold (2%)^35^. Peptides with strong binding or weak binding affinity were defined as neoantigens (Fig. 4). Finally, we got 13 potential AS-derived neoantigens from 10 highly recurrent abnormal isoforms (present in at least 10 UM cases) due to changes in amino acid sequences (Table 3).

**Table 3:** Sequences, binding scores and levels of neoantigens.

Isoform ID	Peptide	Score	Bind Level
Case20_TCONS_00011217	CQVDGLIFL	0.65303	WB
Case20_TCONS_00011217	SLHCQVDGL	0.4821	WB
Case20_TCONS_00118164	WLIHKTTKL	0.59746	WB
Case20_TCONS_00118487	SQADKFLSL	0.51318	WB
Case14_TCONS_00003591	GLFFSHAGV	0.68942	SB
Case14_TCONS_00032920	AILEIGAGV	0.71785	SB
Case14_TCONS_00047054	CVLHELFHL	0.5366	WB
Case14_TCONS_00047054	RILCVLHEL	0.70888	SB
Case14_TCONS_00065906	AFWDWSVEA	0.48128	WB
Case14_TCONS_00066759	KLSHPMVAI	0.63195	WB
Case14_TCONS_00069531	FMRLPLISV	0.64744	WB
Case14_TCONS_00069531	RLPLISVAL	0.54154	WB
Case5_TCONS_00029306	LLAQLGFPL	0.78058	SB

## Data Records

The datasets presented here have been stored at GEO under GSE206464^36^. Our cohort includes a total of 20 cases, of which 20 cases have short-read RNA-seq data and 18 cases have long-read RNA-seq data. This study is based on the 18 cases with both short-read and long-read RNA-seq data. AlphaFold-Multimer predicted structures files are accessible at figshare^37^. Each folder, whose name consists of gene name and isoform ID, corresponds to each isoform structure files (for example, “AAMDC_TCONS_00022447”, which means isoform “TCONS_00022447” of gene AAMDC). Each folder contains multiple format text files which represent the predicted structures information. Among all predicted structures files, “ranked_0.pdb” file has the highest confidence.

## Technical Validation

### Sequencing quality of long-read RNA-seq data

Pychopper package (https://github.com/nanoporetech/pychopper) was used to identify, orient and rescue FL cDNA reads. The number of total long reads ranged from 4,788,440 to 14,048,314 in which the FL reads were between 4,112,595 and 12,828,344 (Table 4). We observed an average of 7.05 million FL reads (accounting for 87.49% of total long reads) with an average read quality of 10.78 (ranging from 9.9 to 12.8), confirming the high confidence in the quality of sequencing data (Table 4).

**Table 4:** Sequencing statistics of 18 UM cases.

Case	Data (Gb)	Total reads	FL reads	Mean read length	Mean read quality
Case 2	2.8	6549553	5623406	1094	12.8
Case 3	8.5	8102441	6632235	1046	10.1
Case 4	7.2	7820006	6670112	918	10
Case 5	3.4	4788440	4215364	691	10.6
Case 6	6.3	10669535	9770237	589	10.5
Case 7	7.8	8210582	7042750	953	10
Case 9	4.8	7326024	6503192	646	10.6
Case 10	3.1	4798635	4112595	1069	12.7
Case 11	6.1	8495028	7596197	706	10.9
Case 12	7.1	7675607	6652587	932	10.8
Case 13	5.3	8593514	7783746	606	10.7
Case 14	4.9	4843576	4127856	994	9.9
Case 15	7.1	7745207	6780170	863	12.8
Case 16	6	13066897	11847478	448	10.2
Case 17	5.3	7943859	7142291	652	10.6
Case 18	9.2	8144175	6649344	1130	10
Case 19	3.7	5510264	4937671	670	10.6
Case 20	6.1	14048314	12828344	423	10.2

## Data Availability

Here we list the details of the software used for data analysis. Pychopper (https://github.com/epi2me-labs/pychopper), version 2, was used to identify, orient and trim FL Nanopore cDNA reads. LoRDEC, version 1.4.1, was used for correcting errors of long-read RNA-seq data based on short-read RNA-seq data. Pinfish (https://github.com/nanoporetech/pinfish), version 0.1.0, which is a collection of tools helping to make sense of long-read RNA-seq data. Flair (https://github.com/BrooksLabUCSC/flair), version 1.5.0, was used for isoform definition with long-read RNA-seq data. Cuffcompare, version 2.2.1, was used to identify novel isoform based on gene annotation information. Samtools, version 1.9, was used to extract sequence according to the coordinates of novel isoforms. ORFfinder, version 0.4.3, was used to predict ORFs based on nucleotide sequences. AlphaFold Multimer (an extension of AlphaFold2, version 2.2.0) was used to predict the 3D structures of novel isoforms. Then, we also use some in-house scripts to filter and prepare the input and output files, which have been deposited in github (https://github.com/ZhangNestor/magic).

## References

[1] Pan Q, Shai O, Lee LJ, Frey BJ, Blencowe BJ (2008). Deep surveying of alternative splicing complexity in the human transcriptome by high-throughput sequencing. Nat Genet.

[2] Jiang W, Chen L (2021). Alternative splicing: Human disease and quantitative analysis from high-throughput sequencing. Comput Struct Biotechnol J.

[3] Chen K, Dai X, Wu J (2015). Alternative splicing: An important mechanism in stem cell biology. World J Stem Cells.

[4] Moore MJ, Wang Q, Kennedy CJ, Silver PA (2010). An alternative splicing network links cell-cycle control to apoptosis. Cell.

[5] Bonnal SC, Lopez-Oreja I, Valcarcel J (2020). Roles and mechanisms of alternative splicing in cancer - implications for care. Nat Rev Clin Oncol.

[6] Zhang Y, Qian J, Gu C, Yang Y (2021). Alternative splicing and cancer: a systematic review. Signal Transduct Target Ther.

[7] Kahles A (2018). Comprehensive Analysis of Alternative Splicing Across Tumors from 8,705 Patients. Cancer Cell.

[8] Alsafadi S (2016). Cancer-associated SF3B1 mutations affect alternative splicing by promoting alternative branchpoint usage. Nat Commun.

[9] Group PTC (2020). Genomic basis for RNA alterations in cancer. Nature.

[10] Climente-Gonzalez H, Porta-Pardo E, Godzik A, Eyras E (2017). The Functional Impact of Alternative Splicing in Cancer. Cell Rep.

[11] Stanley RF, Abdel-Wahab O (2022). Dysregulation and therapeutic targeting of RNA splicing in cancer. Nat Cancer.

[12] Steijger T (2013). Assessment of transcript reconstruction methods for RNA-seq. Nat Methods.

[13] Bolisetty MT, Rajadinakaran G, Graveley BR (2015). Determining exon connectivity in complex mRNAs by nanopore sequencing. Genome Biol.

[14] Tang AD (2020). Full-length transcript characterization of SF3B1 mutation in chronic lymphocytic leukemia reveals downregulation of retained introns. Nat Commun.

[15] Aw JGA (2021). Determination of isoform-specific RNA structure with nanopore long reads. Nat Biotechnol.

[16] Watson M, Warr A (2019). Errors in long-read assemblies can critically affect protein prediction. Nat Biotechnol.

[17] Salmela L, Rivals E (2014). LoRDEC: accurate and efficient long read error correction. Bioinformatics.

[18] Wang JR, Holt J, McMillan L, Jones CD (2018). FMLRC: Hybrid long read error correction using an FM-index. BMC Bioinformatics.

[19] Varadi M (2022). AlphaFold Protein Structure Database: massively expanding the structural coverage of protein-sequence space with high-accuracy models. Nucleic Acids Res.

[20] Sommer, M. J. *et al*. Structure-guided isoform identification for the human transcriptome. *Elife* **11**, 10.7554/eLife.82556 (2022). 10.7554/eLife.82556 PMC9812405 36519529

[21] UniProt C (2021). UniProt: the universal protein knowledgebase in 2021. Nucleic Acids Res.

[22] Armstrong DR (2020). PDBe: improved findability of macromolecular structure data in the PDB. Nucleic Acids Res.

[23] Kuhlman B, Bradley P (2019). Advances in protein structure prediction and design. Nat Rev Mol Cell Biol.

[24] Jumper J (2021). Highly accurate protein structure prediction with AlphaFold. Nature.

[25] Pinheiro F, Santos J, Ventura S (2021). AlphaFold and the amyloid landscape. J Mol Biol.

[26] Li H (2021). New strategies to improve minimap2 alignment accuracy. Bioinformatics.

[27] Vaser R, Sovic I, Nagarajan N, Sikic M (2017). Fast and accurate de novo genome assembly from long uncorrected reads. Genome Res.

[28] Dobin A (2013). STAR: ultrafast universal RNA-seq aligner. Bioinformatics.

[29] Trapnell C (2010). Transcript assembly and quantification by RNA-Seq reveals unannotated transcripts and isoform switching during cell differentiation. Nat Biotechnol.

[30] Li H (2009). The Sequence Alignment/Map format and SAMtools. Bioinformatics.

[31] Rombel IT, Sykes KF, Rayner S, Johnston SA (2002). ORF-FINDER: a vector for high-throughput gene identification. Gene.

[32] Zhang Y, Skolnick J (2004). Scoring function for automated assessment of protein structure template quality. Proteins.

[33] Xu J, Zhang Y (2010). How significant is a protein structure similarity with TM-score = 0.5?. Bioinformatics.

[34] Zhang N (2023). Figshare.

[35] Reynisson B, Alvarez B, Paul S, Peters B, Nielsen M (2020). NetMHCpan-4.1 and NetMHCIIpan-4.0: improved predictions of MHC antigen presentation by concurrent motif deconvolution and integration of MS MHC eluted ligand data. Nucleic Acids Res.

[36] Zhang Z, Shen JF (2022). GEO..

[37] Zhang N (2023). Figshare.

